# Effect of Different Winemaking Conditions on Organic Acids Compounds of White Wines

**DOI:** 10.3390/foods10112569

**Published:** 2021-10-25

**Authors:** Elena Cristina Scutarașu, Iulian Valentin Teliban, Cătălin Ioan Zamfir, Camelia Elena Luchian, Lucia Cintia Colibaba, Marius Niculaua, Valeriu V. Cotea

**Affiliations:** 1 Horticulture Department, Iași University of Life Sciences, 3rd M. Sadoveanu Alley, 700490 Iași, Romania; cristina_scutarasu@yahoo.com (E.C.S.); telibaniulian@yahoo.com (I.V.T.); kamelia_luchian@yahoo.com (C.E.L.); cintia.colibaba@gmail.com (L.C.C.); 2 Research Center for Oenology, Romanian Academy, Iași Branch, 9th M. Sadoveanu Alley, 700490 Iași, Romania; catalin.zamfir@yahoo.com (C.I.Z.); niculaua@gmail.com (M.N.)

**Keywords:** organic acids, yeasts, fermentation, HPLC, Aligoté, wines

## Abstract

Organic acids represent naturally occurring compounds that are found in many types of food and beverages, with important functions in defining products’ final quality. Their proportions in wine are dependent on grape composition and winemaking conditions (temperature, pH levels, oxygen, and carbon dioxide concentration). Therefore, this article studied the influence of different fermentation conditions (200 hL tanks vs. 50 L glass demijohns) and various yeasts on the evolution of the main organic acids during alcoholic fermentation of “Aligoté” wines. The fermentation lasted 22 days and samples were collected daily. Laboratory analyses were quantified according to the International Organization of Vine and Wine recommendations. High-performance liquid chromatography for the identification and quantification of organic acids was used. The data showed the important effect of winemaking conditions on sugar consumption, density or acidity values, and sensory characteristics. Significant differences in organic acid concentrations (especially for tartaric acid) were obtained for all variants, depending on the fermentation conditions, inoculated yeast and the sampling moment. The quantities of most of the identified organic acids were generally significantly increased when glass vessels were used, compared to those fermented in tanks. Most organic acids concentrations were favored by lower pH and showed higher values at lower temperatures.

## 1. Introduction

Wine results from the alcoholic fermentation of grape juice, managed by numerous microorganisms (yeast and bacteria). The supplement of yeast strains from commercial sources optimizes the fermentation process by making it safer and easier to control. Saccharomyces cerevisiae is often used in the winemaking process. During fermentation, yeasts carry out the biotransformation of fruits into wine compounds by converting sugars into ethanol and other diverse metabolites [1].

Organic acids represent naturally occurring compounds produced from the catabolism of amino acids, being intermediates in metabolic pathways. Organic acids are found in many types of food and beverages (juice, coffee, tea, and wine) [2], favoring the capacity of antioxidants to manage free radicals. They also have antibiotic, antimicrobial properties and improve wine stability [3]. The main organic acids identified in wine are tartaric, oxalic, succinic, malic, acetic, and lactic acids. Their origin can be represented by raw materials (above 90% of the total concentration of organic acids in grapes are represented by malic and tartaric acids), or it can result from the yeast metabolism during winemaking [4,5]. Of these, malic, citric and tartaric acids contribute in the highest proportion on wine titratable acidity. This parameter is an elementary factor of important management decisions related to contamination risks and sensorial features. Optimal acidity confers freshness and vivacity to wines, but as the value of this parameter increases, the wines become harsh, difficult to consume [6].

Several authors have confirmed a major contribution of organic acid to wine’s sensory profile [3,7,8,9]. Moreover, succinic, pyruvic, and lactic acids can be correlated with fresh, sour, and even metallic flavor of wines; acetic acid brings a vinegary perception, while succinic acid presents a salty-bitter taste [10]. Moreover, malic acid is important to measure the development of malolactic fermentation; acetic acid is the principal indicator of fermentation problems or spoilage, while citric acid may be supplemented to correct acidity [11]. The separation and quantification of organic acids are of high interest in the quality control of wine as an indicator of degradation due to storage conditions or aging process, or to determine authenticity [12]. Several authors studied the impact of different winemaking conditions on organic acid concentrations, but there are limited data regarding their evolution during fermentation stage. For example, Baiano et al. [13] reported a minor variation of malic acid level obtained during winemaking, tartaric acid content was significantly diminished when using cryomaceration technique compared to traditional technology and citric acid presented upper values. Other authors demonstrated a major influence of temperature, sugar, and pH levels [3] on succinic acid [14]. The formation of acetic acid is significantly affected by the yeast strain [15], sugar level, pH [16,17], or fermentation temperature [3]. In addition, pyruvic acid production is favored by high pH values [18] and the degree of aeration [19]. Regarding Chidi et al. [3], the concentration of citric acid is strongly dependent on oxygen availability in the fermentation vessel.

The importance of determining the content of organic acids in wine is also due to the function it manifests on consumers’ health and promoting moderate wine consumption as an important component of a balanced diet. These compounds bind free radicals in the human body system if found in the diet. For example, most organic acids promote iron absorption in the human body [4] and tartaric acid has positive effects on colon function [20,21]. Regarding succinic acid, important effects of this compound on cardiovascular diseases have been reported [22], while malic acid presents an important role in maintaining gastrointestinal health [23]. In addition, citric acid can meliorate ketosis and has a positive effect against diabetes. Citric and malic acids were confirmed to have a protective role on the myocardium and act on ischemic lesions [4].

Wines’ composition and structure is in continuous evolution and there is a constant necessity for progressive research. The potential variation of yeasts in juice composition is enormous. The capacities to control and efficiently ferment the ever-changing compositional medium of must are primal attributes of commercial oenological yeasts. These properties are dependent on overcoming a range of environmental variables, including oxygen availability, juice clarity, or temperature. Due to the plenty of potential variations in grape juice composition corroborated with a large number of available commercial yeast strains, more studies need to be conducted on these products’ influence [24]. It is also known that the quality of wines is dependent on the variety, terroir, and the particularities of the applied technology.

“Aligoté” is an international grape variety that gained large appreciation in Romania in recent years, but there are not many studies on its composition. Therefore, this article aimed to study the impact of different winemaking conditions on the evolution of the main organic acids during alcoholic fermentation of “Aligoté”wines, comparing the samples obtained in 200 hL stainless steel tanks and with controlled atmospheric conditions (light, temperature) to those obtained in 50 L glass demijohns and uncontrolled atmospheric conditions. Moreover, the effect of different commercial yeasts (*Saccharomyces* spp.) on these compounds was assessed.

## 2. Materials and Methods

### 2.1. Winemaking Process

**Figure 1:**
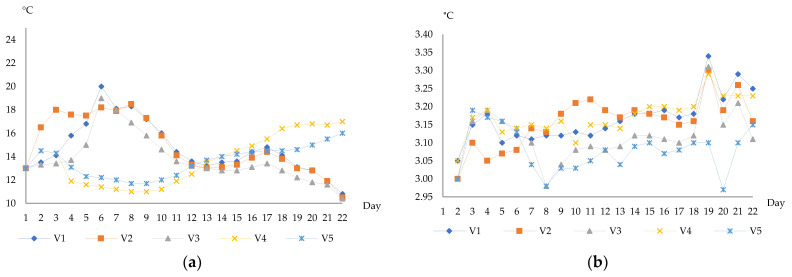
Temperature (**a**) and pH (**b**) levels during the alcoholic fermentation of analyzed samples.

For this experiment, the Aligoté variety was selected, being widely cultivated in Iasi vineyards. The grapes were processed in the autumn of 2018 according to the classic technology of obtaining white wines. The grape juice was initially collected in 5 stainless steel tanks for yeast inoculation and homogenization, and the resulted mixture was divided into 50 L glass vessels and 200 hL stainless steel tanks, respectively. Four different Saccharomyces yeasts often used in winemaking were analyzed. The samples were registered as follows: V1—no yeast, fermented in glass vessels; V2—Fermol^®^ Candy, fermented in glass vessels; V3—Fermactive^®^ Sauvignon, fermented in glass vessels; V4—Fermol^®^ Candy, fermented in stainless steel tanks; V5—Fermactive^®^ Sauvignon, fermented in stainless steel tanks. Throughout the alcoholic fermentation, the pH level and temperature conditions were measured (Figure 1). The alcoholic fermentation was stopped after 22 days for all variants. Samples collected daily were kept at −20 °C for 6 months.

### 2.2. Methods of Analysis

Basic physicochemical parameters (Table 1) of resulting wines were followed according to the International Organization of Vine and Wine instructions [25]: titratable acidity(g/L tartaric acid); volatile acidity (g/L acetic acid) using titrimetric measures; ethanol (% vol.), with a Dujardin-Salleron D.E. 2000 machine—for distillation; pH and density (instrumental measurements) and sugar (g/L), by the Luff-Schoorl method.

Identification and determination of main organic acids was performed according to International Organization of Vine and Wine (OIV) recommendations [25], using a Shimadzu LC-DAD series 20 system (Shimadzu Scientific Instruments Inc., Columbia, MD, USA) and coupled with a UV spectrophotometer (Shimadzu Scientific Instruments Inc., Columbia, MD, USA). Malic, tartaric and succinic acids were determined using two columns (L = 250 mm and d_internal_ = 4 mm) (Shimadzu Scientific Instruments Inc., Columbia, Maryland, USA), fitted with octyl-bonded silica (d = 5 µm, with spherical particles), while for citric, acetic, and lactic acids, an ion exchange resin column (with H^+^ model, L = 300 mm, d_internal_ = 7.8 mm and 9 µm particle size) (Shimadzu Scientific Instruments Inc., Columbia, MD, USA) were used. Regarding the determination of citric, lactic, and acetic acids, the mobile phase was represented by a solution of sulfuric acid 0.0125 mol/L. The elution flow was 0.6 mL/min, at 60–65 °C. For the determination of succinic, malic, and tartaric acids, the mobile phase was mono-potassium phosphate (60 g/L) and ammonium sulfate (14 g/L), adjusted to pH 2.1 by the addition of phosphoric acid. In this case, the elution rate was 0.8 mL/min, at 20 °C.

The samples were filtered using sterile 0.45 μm filters. A volume of 8 mL of the filtered sample was injected into the SPE cartridge (previously washed with 10 mL of methanol and then with 10 mL of water). 10 μL of reference solution and 10 μL of working solution were injected successively into the HPLC system. Each organic acid corresponds to an absorption spectrum. All samples were analyzed in triplicate.

Sensory characteristics of final samples were evaluated by a professional panel of 12 tasters, 6 men and 6 women (represented by winemakers, laboratory personal, and researchers). The samples’ sensory quality was appreciated by defining aroma descriptors, as shown in the Results and Discussion section. The odor intensity of the analyzed parameters was evaluated by means of a hedonistic scale from 0 (absence) to 10 (maximum).

Statistical test made possible to establish the existence of homogeneous groups and significant differences. Multivariate analysis was done using Statgraphics Centurion, version 18 (Statgraphics Technologies Inc., The Plains, VA, USA). All results were analyzed in triplicate and presented as mean plus standard deviation. Correlation analysis for the final samples (last day of sampling) was performed using Displayr software (Displayr Australia Pty Limited, Glebe, Australia), while sensory data representation was performed with Excel package (Microsoft Corporation, Redmond, WA, USA).

Reagents. All reagents and standards were of analytical grade and supplied by Merck KgaA (Darmstadt, Germany): methanol (PubChem CID: 887); tartaric acid (PubChem CID: 875); malic acid (PubChem CID: 525); sodium lactate (PubChem CID: 23666456); sodium acetate (PubChem CID: 517045); succinic acid (PubChem CID: 1110); citric acid (PubChem CID: 311); sulphuric acid (*ρ*_20_ = 1.84 g/mL) (PubChem CID: 1118); sulphuric acid solution (0.0125 mol/L); dipotassium hydrogen *o*-phosphate (PubChem CID: 24450); ammonium sulphate (PubChem CID: 6097028); *o*-phosphoric acid, (85%, *ρ_20_* = 1.71 g/mL) (PubChem CID: 1004).

## 3. Results and Discussion

### 3.1. Physicochemical Characteristics of Wines

The main physicochemical characteristics of the final samples are presented in Table 1. The pH level and temperature conditions can be observed in Figure 1. The type of analyzed yeasts manifested only minor modifications on most basic parameters, whose levels are within the limits allowed by OIV recommendations [25]. Significant differences could be observed on sugar levels, with values ranging from 2.4 g/L (V2) to 6.3 g/L (V1), showing a different capacity of inoculated yeasts to complete fermentation. In accordance with sugar levels, total acidity and density presented significant differences between variants. Considering the level of residual sugars in the final samples, V1 was a semi-dry wine, while the rest of the samples were dry ones.

Previous studies [26,27,28] showed similar results on sparkling wines and are in accordance with other literature data [29].

### 3.2. Organic Acid Concentrations

Data showed significant differences regarding the above-mentioned compounds (Table 2, Table 3, Table 4, Table 5 and Table 6), according to the fermentation stage, environmental conditions, or inoculated yeasts.

Tartaric acid represents the main contributor to wine acidity and manifests an important role in its organoleptic perception [30]. The concentrations of tartaric acid show various oscillations during the alcoholic phase, depending on the sampling moment. A significantly (*p* < 0.05) increased concentration of tartaric acid can be observed in the primary phase of the fermentation in all variants, followed by a constant decrease until the end of the process. In agreement with Waterhouse et al. [31], although tartaric acid is not metabolized during winemaking, it can be lost through physicochemical mechanisms, such as accumulation of ethanol or neutralization by cations (for example, K^+^, Ca^2+^, Na^+^). This compound can be found in ionizing and non-ionizing form or salts [32]. Tartaric acid degradation is also related to yeast species [33] and fungi [34]. Final samples presented different concentrations of tartaric acid, according to the inoculated yeast, the highest value being registered in the V3 sample (3.97 g/L). The lowest values of tartaric acid were identified in V4 and V5, in which the fermentation took place in tanks, which may be due to a considerable increase in ethanol during the fermentation stage, combined with low fermentation temperature. Comparable values of tartaric acid were obtained by Moroșanu et al. [27]. Bayraktar [10] reported a positive correlation between tartaric acid concentration and citric acid in wine.

The importance of determining malic acid in wine can be derived from high antibacterial activity, due to synergistic effects of the organic acids (especially malic and tartaric acids), alcohol, and acidic pH, respectively [3]. According to Vilela [35], *Saccharomyces* yeasts cannot efficiently degrade malic acid during alcoholic fermentation. In the first phase of the fermentation process, there is a conversion of small quantities of sugar into alcohol (approximately 2% vol.), corroborated by a reduction of malic acid concentration and production of various secondary products. Amerine and Kunkee [36] reported a significant decrease in malic acid concentrations when Schizosaccharomyces pombe is present. From another point of view, de Klerk [37] postulated that malic acid could be converted in succinic acid in high proportions. The conversion of malic into lactic acid in wine conducts to a significant modification in pH and wines flavors. For all these reasons, only small concentrations of malic acid can be found in finished wines [5]. Regarding the obtained results, V1 and V2 variants (1.56 g/L) recorded the highest concentration of malic acid at the end of the fermentation, while V4 sample presented the smallest value (1.21 g/L). These results are in agreement with Whiting’s [38], who reports high values of malic acids when winemaking conditions were uncontrolled.

Acetic acid is mostly produced during the yeast growth phase. Its concentration in wine depends on many factors, including fermentation temperature, chemical composition of raw materials (e.g., sugar concentrations, minerals, vitamins, nitrogen, and structure of microorganisms in the environment) [33]. Regarding the analyzed wines, important concentrations were reported at the end of the fermentation. This can be explained by a defective pyruvate decarboxylase activity [33] and by the increase of the temperature in the second part of the fermentation phase. According to Ugliano and Henschke [39], the most important factor for controlling acetic acid content during fermentation is the yeast strain used. Following the analysis of the results obtained by HPLC, the V3 sample (0.26 g/L) presented the highest concentration of acetic acid, while the lowest content was recorded in the V4 sample (0.19 g/L). Comparable amounts of acetic acid were reported by Bely et al. [16]. The different values of acetic acid in the fermented variants in small capacity glass vessels (50 L) with different yeasts can be explained by the high intraspecific variability of the Saccharomyces species strains yeast.

Citric acid content is correlated to the formation of diacetyl and acetate, and has an essential influence on wine aroma and its stability [33]. The concentration of this compound showed an upward trend during fermentation. The citric acid level of the samples was between 0.21 g/L (V1 and V4) and 0.23 g/L (V2), being significantly influenced by the inoculated yeasts. The importance of monitoring the citric acid level is derived from its antioxidant and antimicrobial properties. Also, citric acid is involved in the metabolism of most microorganisms, being an essential mediator in the tricarboxylic acid cycle [40].

Succinic acid is considered the primary contributor of titratable acidity, representing about 90% of the non-volatile acids resulting during must fermentation, which confers a salty and slightly bitter taste in the wine. This compound can result from the oxidative decarboxylation of the Krebs cycle or from the glyoxylate cycle, or by the decarboxylation of α-ketoglutaric acid under the influence of oxidizing factors [41]. Regarding the analyzed samples, the concentrations of succinic acid showed a constant increase during fermentation. Its production is usually influenced by the yeast assimilable nitrogen, and temperature (<18 °C) (Figure 1). The highest content of succinic acid was identified in V3 variant (0.34 g/L), while the lowest level was registered in V2 (0.32 g/L). Similar results were presented by Margalit [42].

Lactic acid is generally produced by yeast during alcoholic fermentation [43], and its determination is important not only for essential functions in wine quality, but also due to its health benefits, such as better digestion of lactose, with favorable effect against cancer, and in maintaining cholesterol levels [44]. Its concentration presented significant increases, according to the fermentation stage and inoculated yeasts. Regarding the final samples, the highest value was recorded in the V3 variant (0.34 g/L), followed by V1 (0.18 g/L). These higher values can be correlated with the ability of inoculated yeast to synthesize a larger amount of lactic acid during malolactic fermentation. Zotou et al. [5] reported a descendent concentration of this compound during alcoholic fermentation when Acetobacter and Gluconobacter species were present, confirming the significant effect of different bacteria species on the organic acid profile.

**Figure 2:**
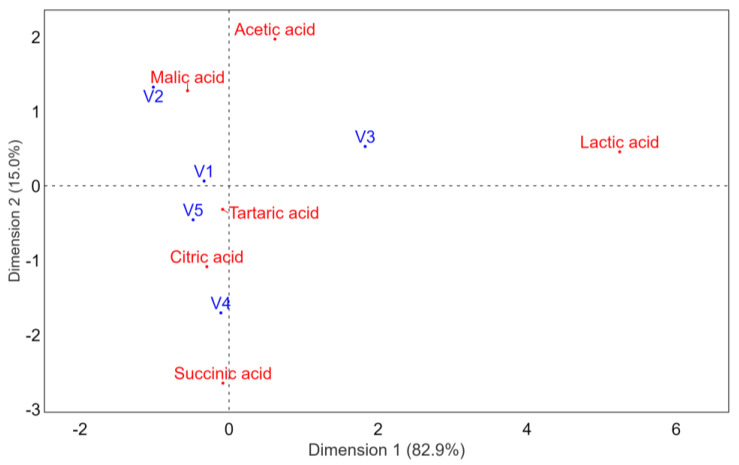
Correspondence analysis of wine’s organic acids profile.

From Figure 2, different organic acids profile of final samples can be observed, depending on the existing variables.

### 3.3. Sensory Description

**Figure 3:**
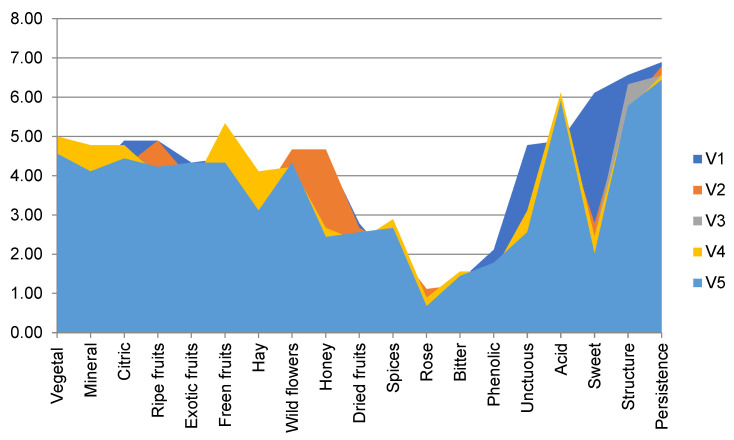
Sensory perception of analyzed samples.

Figure 3 presents the sensory profile of analyzed wines. Data showed a different sensory character of analyzed samples, depending on the applied winemaking conditions. All experimental samples were appreciated as equilibrated, with good acidity, structure and persistence.

The V1 variant was described by high notes of citric, honey and ripe fruits odor, sweet taste and unctuous texture but lower acidity. Floral odor (wild flowers and rose) better characterized the V2 variant. The higher acidity was appreciated in the V4 sample, with vegetal and green fruits notes and spiced taste.

Significant differences (Table 2, Table 3, Table 4, Table 5 and Table 6) were obtained for the final concentration of organic acids in the majority of identified compounds (*p* < 0.05). The quantities of most organic acids were generally significantly higher under uncontrolled conditions (fermented in glass vessels) compared to those fermented in tanks. These observations are in agreement with literature data [8,45]. Identified compounds showed higher values at lower temperatures through the fermentation process (Figure 1). The V3 variant showed a significant increase in most of the studied compounds. According to Pan et al. [46], pH represents a strong mediator of metabolic reactions in lactic acid bacteria in wine, including pyruvate synthesis and the production of many metabolites. Tartaric, citric, malic, succinic, and acetic acid concentrations were favored by lower pH, while the lactic acid showed small values on higher pH. According to Samoticha et al. [1], these results indicate good stability to microbial spoilage and stable coloration. The pH level of the analyzed samples can also indicate the existence of both dissociated and non-dissociated forms of some acids, such as tartaric, lactic, and malic acids. In all tested wines, tartaric and citric acids were dominant, similar to the results reported by Torrens et al. [47]. In accordance to Picariello et al. [48], the climate changes can manifest important variations of grapes’ organic acids content.

From Figure 2 and Figure 3, different organic acids and sensory profiles of final samples can be remarked, depending on the inoculated yeast and fermentation conditions.

Due to the considerable content in organic acids, wines can be important components in ensuring a balanced diet. This study contributes to the enrichment of literature data and to the optimization of winemaking techniques.

## 4. Conclusions

Different winemaking conditions generated considerable variations of some parameters considered essential in defining wine’s final quality. Significant differences in sugar consumption, density, and acidity were observed. Moreover, different winemaking conditions generated important variations of sensory characteristics. The production of organic acids is significantly influenced the fermentation conditions. The quantities of most of the identified organic acids were generally significantly increased under uncontrolled conditions when glass vessels were used, compared to those fermented in tanks and under controlled conditions. An important contribution to wine’s final quality is attributed to yeast type. Tartaric acid content showed significant differences for all analyzed wines. The V3 variant was remarkable for its highest concentrations in tartaric, acetic, and succinic acids, while the blank sample was characterized by high levels in lactic and malic acids. pH and temperature values show important contributions in organic acid formation. Tartaric, citric, malic, succinic, and acetic acid concentrations were favored by lower pH, while lactic acid showed low values on higher pH levels. Identified compounds showed higher values at lower temperatures.

## Figures and Tables

**Table 1:** Main physicochemical parameters of the samples after the end of the alcoholic fermentation.

Variant	Volatile Acidity (g/L C_2_H_4_O_2_)	Total Acidity (g/L C_4_H_6_O_6_)	pH	Density	Alcoholic Strength (% vol.)	Sugar (g/L)
V1	0.38 ± 0.00	6.47 ± 0.00 *	3.00 ± 0.00	0.9940 ± 0.00 *	11.00 ± 0.01	6.30 ± 0.00 *
V2	0.32 ± 0.00	6.88 ± 0.00 *	3.09 ± 0.00	0.9918 ± 0.00 *	11.20 ± 0.00	2.40 ± 0.01 *
V3	0.29 ± 0.00	6.89 ± 0.00 *	3.04 ± 0.00	0.9928 ± 0.00 *	11.20 ± 0.00	3.60 ± 0.00 *
V4	0.29 ± 0.00	5.92 ± 0.00 *	3.02 ± 0.00	0.9927 ± 0.00 *	11.50 ± 0.00	3.50 ± 0.01 *
V5	0.28 ± 0.00	6.26 ± 0.00 *	3.02 ± 0.00	0.9925 ± 0.00 *	11.30 ± 0.00	3.30 ± 0.00 *
*p*-value	ns	*p* < 0.05	ns	*p* < 0.05	ns	*p* < 0.05

V1—no yeast, fermented in glass vessels. V2—Fermol^®^ Candy, fermented in glass vessels. V3—Fermactive^®^ Sauvignon, fermented in glass vessels. V4—Fermol^®^ Candy, fermented in stainless steel tanks. V5—Fermactive^®^ Sauvignon, fermented in stainless steel tanks. Samples were analyzed in triplicate and values are presented as mean and obtained standard deviation. * indicates significant differences between samples (*p* < 0.05).

**Table 2:** Evolution of main organic acids during alcoholic fermentation of V1 variant (g/L).

Fermentation Stage	Tartaric Acid	Malic Acid	Lactic Acid	Acetic Acid	Citric Acid	Succinic Acid
Day 1 (a)	5.07 ± 0.02 ^efg^	1.87 ±0.02 *	0.10 ± 0.00 *	0.10 ± 0.00 *	0.20 ± 0.00 *	0.26 ± 0.00 *
Day 2 (b)	5.12 ± 0.0 ^cde^	1.83 ± 0.02 *	0.11 ± 0.00 *	0.11 ± 0.00 *	0.20 ± 0.00 *	0.26 ± 0.00 *
Day 3 (c)	5.15 ±0.00 ^b^	1.74 ± 0.01 *	0.14 ± 0.00 *	0.12 ±0.00 ^d^	0.20 ± 0.00 ^d^	0.26 ± 0.00 *
Day 4 (d)	5.11 ±0.00 ^be^	1.70 ± 0.01 *	0.14 ± 0.00 *	0.13 ± 0.00 ^ce^	0.20 ± 0.00 ^ce^	0.27 ± 0.00 ^e^
Day 5 (e)	5.09 ± 0.00 ^abdf^	1.66 ± 0.02 *	0.15 ± 0.00 ^f^	0.13 ±0.00 ^d^	0.20 ± 0.00 ^d^	0.27 ± 0.00 ^df^
Day 6 (f)	5.064 ±0.00 ^aeg^	1.621 ±0.01 *	0.148 ±0.00 ^eg^	0.13 ± 0.00 ^g^	0.204 ±0.00 ^gh^	0.27 ± 0.00 ^e^
Day 7 (g)	5.05 ± 0.01 ^afh^	1.60 ± 0.004 ^h^	0.15 ± 0.00 ^fhi^	0.13 ± 0.00 ^fh^	0.20 ± 0.00 ^fh^	0.27 ± 0.00 *
Day 8 (h)	5.03 ± 0.01 ^gi^	1.59 ± 0.00 ^g^	0.15 ± 0.00 ^gi^	0.13 ± 0.00 ^gij^	0.21 ± 0.00 ^fgi^	0.28 ± 0.00 *
Day 9 (i)	5.00 ± 0.01 ^h^	1.57 ± 0.00 *	0.15 ± 0.00 ^ghj^	0.131 ±0.00 ^hj^	0.21 ± 0.00 ^hj^	0.29 ± 0.00 *
Day 10 (j)	4.95 ± 0.00 *	1.56 ± 0.00 *	0.15 ± 0.00 ^ik^	0.13 ± 0.00 ^hi^	0.21 ± 0.00 ^ik^	0.29 ± 0.00 ^k^
Day 11 (k)	4.88 ±0.03 *	1.54 ± 0.00 ^l^	0.15 ± 0.00 ^jl^	0.13 ± 0.00 *	0.21 ± 0.00 ^jlm^	0.29 ± 0.00 ^j^
Day 12 (l)	4.67 ± 0.03 *	1.53 ± 0.00 ^km^	0.15 ± 0.00 ^km^	0.14 ± 0.00 *	0.21 ± 0.001 ^km^	0.30 ± 0.00 *
Day 13 (m)	4.59 ± 0.03 *	1.51 ± 0.00 ^ln^	0.16 ± 0.00 ^l^	0.14 ± 0.00 *	0.21 ± 0.00 ^kln^	0.30 ±0.00 *
Day 14 (n)	4.46 ±0.02 *	1.50 ± 0.00 ^mo^	0.16 ± 0.00 *	0.14 ± 0.00 *	0.201 ± 0.00 ^mo^	0.30 ± 0.00 *
Day 15 (o)	4.40 ± 0.02 *	1.49 ± 0.00 ^np^	0.16 ± 0.00 *	0.14 ± 0.00 *	0.21 ± 0.00 ^npq^	0.30 ± 0.00 ^p^
Day 16 (p)	4.23 ± 0.04 *	1.48 ± 0.00 ^oq^	0.16 ± 0.00 ^q^	0.15 ± 0.00 *	0.21 ± 0.00 ^oq^	0.31 ± 0.00 ^oq^
Day 17 (q)	4.16 ± 0.02 *	1.47 ± 0.00 ^pr^	0.17 ± 0.00 ^p^	0.15 ± 0.00 *	0.21 ± 0.00 ^opr^	0.31 ± 0.00 ^p^
Day 18 (r)	4.10 ± 0.02 *	1.46 ± 0.00 ^qs^	0.17 ± 0.00 *	0.17 ± 0.00 *	0.21 ± 0.00 ^q^	0.31 ± 0.00 ^s^
Day 19 (s)	4.01 ± 0.04 *	1.45 ± 0.00 ^rt^	0.17 ± 0.00 *	0.18 ± 0.00 *	0.21 ± 0.00 ^t^	0.31 ± 0.00 ^r^
Day 20 (t)	3.92 ± 0.03 *	1.44 ± 0.00 ^su^	0.18 ± 0.00 *	0.18 ± 0.00 *	0.21 ± 0.00 ^s^	0.31 ± 0.00 *
Day 21 (u)	3.86 ± 0.02 *	1.43 ± 0.00 ^tv^	0.18 ± 0.00 *	0.19 ± 0.00 *	0.21 ± 0.00 *	0.32 ± 0.00 *
Day 22 (v)	3.80 ± 0.03 *	1.42 ± 0.00 ^u^	0.18 ± 0.00 *	0.20 ± 0.00 *	0.21 ± 0.00 *	0.32 ± 0.00 *

V1—no yeast, fermented in glass vessels. V2—Fermol^®^ Candy, fermented in glass vessels. V3—Fermactive^®^ Sauvignon, fermented in glass vessels. V4—Fermol^®^ Candy, fermented in stainless steel tanks. V5—Fermactive^®^ Sauvignon, fermented in stainless steel tanks. C—identified compounds. 1—tartaric acid. 2—malic acid. 3—lactic acid. 4—acetic acid. 5—citric acid. 6—succinic acid. Different superscript letters show that the fermentation moments do not have a statistically significant influence on organic acid concentration. *—indicates that the concentration of the identified organic acid is significantly influenced by the fermentation stage.

**Table 3:** Evolution of main organic acids during alcoholic fermentation of V2 variant(g/L).

Fermentation Stage	Tartaric Acid	Malic Acid	Lactic Acid	Acetic Acid	Citric Acid	Succinic Acid
Day 1 (a)	5.07 ± 0.02 *	1.87 ± 0.02 *	0.10 ± 0.00 ^b^	0.10 ± 0.00 *	0.20 ± 0.00 *	0.26 ± 0.00 ^b^
Day 2 (b)	5.19 ± 0.02 *	1.84 ± 0.00 *	0.10 ± 0.00 ^a^	0.11 ± 0.00	0.20 ± 0.00 *	0.26 ± 0.00 ^a^
Day 3 (c)	5.12 ± 0.05 *	1.82 ± 0.00 *	0.13 ± 0.00 ^de^	0.14 ± 0.00 ^de^	0.21 ± 0.00 ^de^	0.26 ± 0.00 *
Day 4 (d)	5.63 ± 0.04 *	1.80 ± 0.00 *	0.13 ± 0.00 ^cef^	0.14 ± 0.00 ^ce^	0.21 ± 0.00 ^cefg^	0.27 ± 0.00 *
Day 5 (e)	5.29 ± 0.02 *	1.75 ± 0.01 *	0.13 ± 0.00 ^cdfg^	0.14 ± 0.00 ^cd^	0.21 ± 0.00 ^cdfghij^	0.27 ± 0.00 *
Day 6 (f)	5.24 ± 0.02 *	1.72 ± 0.01 *	0.13 ± 0.00 ^degh^	0.15 ± 0.00 ^g^	0.21 ± 0.00 ^deghijk^	0.28 ± 0.00 *
Day 7 (g)	4.89 ± 0.05 *	1.69 ± 0.00 *	0.13 ± 0.00 ^efhi^	0.16 ± 0.00 ^fh^	0.21 ± 0.00 ^defhijkl^	0.28 ± 0.00 *
Day 8 (h)	4.78 ± 0.00 *	1.67 ± 0.00 *	0.13 ± 0.00 ^fgij^	0.16 ± 0.00 ^gi^	0.21 ± 0.00 ^efgijklm^	0.29 ± 0.00 *
Day 9 (i)	4.63 ± 0.04 *	1.65 ± 0.00 *	0.13 ± 0.00 ^ghjk^	0.16 ± 0.00 ^hj^	0.21 ± 0.00 ^efghjklmn^	0.29 ± 0.00 *
Day 10 (j)	4.52 ± 0.03 *	1.64 ± 0.00 ^k^	0.13 ± 0.00 ^hiklm^	0.16 ± 0.00 ^ik^	0.21 ± 0.00 ^efghiklmno^	0.30 ± 0.00 ^k^
Day 11 (k)	4.48 ± 0.01 ^l^	1.63 ± 0.00 ^jl^	0.13 ± 0.00 ^ijlm^	0.16 ± 0.00 ^j^	0.21 ± 0.00 ^fghijlmnop^	0.30 ± 0.00 ^jl^
Day 12 (l)	4.44 ± 0.01 ^km^	1.62 ± 0.00 ^kmn^	0.14 ± 0.00 ^jkmn^	0.17 ± 0.00 ^m^	0.21 ± 0.00 ^ghijkmnop^	0.30 ± 0.00 ^km^
Day 13 (m)	4.40 ± 0.00 ^ln^	1.62 ± 0.00 ^ln^	0.14 ± 0.00 ^jklno^	0.18 ± 0.00 ^ln^	0.21 ± 0.00 ^hijklnopq^	0.30 ± 0.00 ^ln^
Day 14 (n)	4.37 ± 0.02 ^m^	1.61 ± 0.00 ^lmo^	0.14 ± 0.00 ^lmop^	0.18 ± 0.00 ^m^	0.22 ± 0.00 ^ijklmopq^	0.30 ± 0.00 ^mo^
Day 15 (o)	4.31 ± 0.00 ^p^	1.61 ± 0.00 ^npq^	0.14 ± 0.00 ^mnp^	0.19 ± 0.00 *	0.22 ± 0.00 ^jklmnpqr^	0.30 ± 0.00 ^np^
Day 16 (p)	4.27 ± 0.00 ^oq^	1.60 ± 0.00 ^oqr^	0.14 ± 0.00 ^noq^	0.19 ± 0.00 *	0.22 ± 0.00 ^klmnoqrs^	0.31 ± 0.00 ^oq^
Day 17 (q)	4.24 ± 0.00 ^pr^	1.60 ± 0.00 ^opr^	0.14 ± 0.00 ^pr^	0.19 ± 0.00 *	0.22 ± 0.00 ^mnoprs^	0.31 ± 0.00 ^pr^
Day 18 (r)	4.20 ± 0.00 ^qs^	1.59 ± 0.00 ^pqst^	0.14 ± 0.00 ^qs^	0.20 ± 0.00 ^*^	0.22 ± 0.00 ^opqs^	0.31 ± 0.00 ^qs^
Day 19 (s)	4.16 ± 0.00 ^rt^	1.59 ± 0.00 ^rtu^	0.14 ± 0.00 ^rtv^	0.20 ± 0.00 *	0.22 ± 0.00 ^pqr^	0.31 ± 0.00 ^rt^
Day 20 (t)	4.13 ± 0.02 ^s^	1.58 ± 0.00 ^rsu^	0.15 ± 0.00 ^suv^	0.21 ± 0.00 *	0.22 ± 0.00 *	0.31 ± 0.00 ^s^
Day 21 (u)	4.08 ± 0.02 *	1.58 ± 0.01 ^st^	0.15 ± 0.00 ^tv^	0.22 ± 0.00 *	0.23 ± 0.00 ^v^	0.32 ± 0.00 ^v^
Day 22 (v)	3.88 ± 0.03 *	1.56 ± 0.01 *	0.15 ± 0.01 ^stu^	0.24 ± 0.00 *	0.23 ± 0.00 ^u^	0.32 ± 0.00 ^u^

V1—no yeast, fermented in glass vessels. V2—Fermol^®^ Candy, fermented in glass vessels. V3—Fermactive^®^ Sauvignon, fermented in glass vessels. V4—Fermol^®^ Candy, fermented in stainless steel tanks. V5—Fermactive^®^ Sauvignon, fermented in stainless steel tanks. Different superscript letters show that the fermentation moments do not have a statistically significant influence on organic acid concentration. *—indicates that the concentration of the identified organic acid is significantly influenced by the fermentation stage.

**Table 4:** Evolution of main organic acids during alcoholic fermentation of V3 variant (g/L).

Fermentation Stage	Tartaric Acid	Malic Acid	Lactic Acid	Acetic Acid	Citric Acid	Succinic Acid
Day 1 (a)	5.07 ± 0.02 ^f^	1.87 ± 0.02 *	0.10 ± 0.00 *	0.10 ± 0.00 ^bc^	0.20 ± 0.00 ^b^	0.26 ± 0.00 *
Day 2 (b)	5.24 ± 0.03 ^c^	1.85 ± 0.00 *	0.13 ± 0.03 *	0.10 ± 0.00 ^ac^	0.20 ± 0.00 ^a^	0.27 ± 0.00 *
Day 3 (c)	5.21 ± 0.02 ^bd^	1.83 ± 0.00 ^d^	0.20 ± 0.00 *	0.10 ± 0.00 ^ab^	0.20 ± 0.00 *	0.28 ± 0.00 *
Day 4 (d)	5.17 ± 0.00 ^ce^	1.82 ± 0.01 ^c^	0.28 ± 0.00 *	0.11 ± 0.00 *	0.21 ± 0.00 *	0.28 ± 0.00 *
Day 5 (e)	5.14 ± 0.02 ^d^	1.80 ± 0.01 *	0.31 ± 0.00 ^fghijkl^	0.16 ± 0.00 *	0.21 ± 0.00 ^f^	0.30 ± 0.00 *
Day 6 (f)	5.09 ± 0.03 ^a^	1.77 ± 0.02 *	0.31 ± 0.00 ^eghijklm^	0.17 ± 0.00 *	0.21 ± 0.00 ^eg^	0.31 ± 0.00 *
Day 7 (g)	5.00 ± 0.05 *	1.71 ± 0.02 *	0.31 ± 0.00 ^efhijklm^	0.18 ± 0.00 *	0.21 ± 0.00 ^fh^	0.32 ± 0.00 ^hi^
Day 8 (h)	4.90 ± 0.05 *	1.61 ± 0.01 *	0.31 ± 0.00 ^efgijklmn^	0.19 ± 0.00 *	0.21 ± 0.00 ^gi^	0.32 ± 0.00 ^gi^
Day 9 (i)	4.77 ± 0.02 *	1.59 ± 0.00 *	0.31 ± 0.00 ^efghjklmno^	0.19 ± 0.00 *	0.21 ± 0.00 ^hj^	0.32 ± 0.00 ^ghjk^
Day 10 (j)	4.71 ± 0.02 ^k^	1.57 ± 0.01 *	0.31 ± 0.00 ^efghiklmno^	0.20 ± 0.00 *	0.21 ± 0.00 ^ik^	0.32 ± 0.00 ^ikl^
Day 11 (k)	4.66 ± 0.02 ^j^	1.54 ± 0.01 *	0.32 ± 0.00 ^efghijlmnop^	0.21 ± 0.00 ^l^	0.21 ± 0.00 ^jl^	0.32 ± 0.00 ^ijl^
Day 12 (l)	4.60 ± 0.02 *	1.51 ± 0.00 ^m^	0.32 ± 0.00 ^efghijkmnopq^	0.21 ± 0.00 ^km^	0.22 ± 0.00 ^km^	0.32 ± 0.00 ^jkm^
Day 13 (m)	4.54 ± 0.03 *	1.50 ± 0.00 ^ln^	0.32 ± 0.00 ^fghijklnopqr^	0.21 ± 0.00 ^ln^	0.22 ± 0.00 ^ln^	0.32 ± 0.00 ^lno^
Day 14 (n)	4.47 ± 0.03 *	1.49 ± 0.00 ^mo^	0.32 ± 0.00 ^hijklmopqrs^	0.21 ± 0.00 ^m^	0.22 ± 0.00 ^mo^	0.32 ± 0.00 ^mo^
Day 15 (o)	4.41 ± 0.02 *	1.48 ± 0.00 ^np^	0.32 ± 0.00 ^ijklmnpqrst^	0.22 ± 0.00 ^p^	0.22 ± 0.00 ^np^	0.32 ± 0.00 ^mnp^
Day 16 (p)	4.35 ± 0.02 *	1.47 ± 0.00 ^oqr^	0.33 ± 0.00 ^klmnoqrst^	0.22 ± 0.00 ^oq^	0.22 ± 0.00 ^oq^	0.33 ± 0.00 ^oq^
Day 17 (q)	4.30 ± 0.03 *	1.47 ± 0.00 ^prs^	0.33 ± 0.00 ^lmnoprstu^	0.22 ± 0.00 ^p^	0.22 ± 0.00 ^pr^	0.33 ± 0.00 ^p^
Day 18 (r)	4.26 ± 0.01 ^s^	1.46 ± 0.00 ^pqst^	0.33 ± 0.00 ^mnopqstu^	0.23 ± 0.00 *	0.22 ± 0.00 ^qs^	0.33 ± 0.00 *
Day 19 (s)	4.22 ± 0.02 ^rt^	1.45 ± 0.00 ^qrtu^	0.33 ± 0.00 ^nopqrtu^	0.23 ± 0.00 *	0.22 ± 0.00 ^rt^	0.33 ± 0.00 *
Day 20 (t)	4.18 ± 0.02 ^s^	1.45 ± 0.00 ^rsu^	0.33 ± 0.00 ^opqrsuv^	0.23 ± 0.00 *	0.22 ± 0.00 ^s^	0.34 ± 0.00 *
Day 21 (u)	4.05 ± 0.04 *	1.44 ± 0.00 ^stv^	0.34 ± 0.00 ^qrstv^	0.25 ± 0.00 *	0.22 ± 0.00 *	0.34 ± 0.00 *
Day 22 (v)	3.97 ± 0.04 *	1.43 ± 0.00 ^u^	0.34 ± 0.00 ^tu^	0.26 ± 0.00 *	0.23 ± 0.00 *	0.34 ± 0.00 *

V1—no yeast, fermented in glass vessels. V2—Fermol^®^ Candy, fermented in glass vessels. V3—Fermactive^®^ Sauvignon, fermented in glass vessels. V4—Fermol^®^ Candy, fermented in stainless steel tanks. V5—Fermactive^®^ Sauvignon, fermented in stainless steel tanks. Different superscript letters show that the fermentation moments do not have a statistically significant influence on organic acid concentration. *—indicates that the concentration of the identified organic acid is significantly influenced by the fermentation stage.

**Table 5:** Evolution of main organic acids during alcoholic fermentation of V4 variant (g/L).

Fermentation Stage	Tartaric Acid	Malic Acid	Lactic Acid	Acetic Acid	Citric Acid	Succinic Acid
Day 1 (a)	5.07 ± 0.02 ^d^	1.87 ± 0.02 *	0.10 ± 0.00 *	0.10 ± 0.00 *	0.20 ± 0.00 ^b^	0.26 ± 0.00 ^b^
Day 2 (b)	5.22 ± 0.03 *	1.82 ± 0.02 *	0.10 ± 0.00 *	0.10 ± 0.00 *	0.20 ± 0.00 ^a^	0.26 ± 0.00 ^a^
Day 3 (c)	5.14 ± 0.01 *	1.51 ± 0.00 *	0.13 ± 0.00 *	0.10 ± 0.00 *	0.20 ± 0.00 *	0.26 ± 0.00 *
Day 4 (d)	5.07 ± 0.04 ^a^	1.49 ± 0.01 *	0.15 ± 0.00 *	0.11 ± 0.00 *	0.20 ± 0.00 *	0.26 ± 0.00 *
Day 5 (e)	4.90 ± 0.02 *	1.39 ± 0.01 *	0.15 ± 0.00 ^f^	0.11 ± 0.00 *	0.21 ± 0.00 *	0.27 ± 0.00 *
Day 6 (f)	4.82 ± 0.02 *	1.36 ± 0.00 *	0.15 ± 0.00 ^eg^	0.11 ± 0.00 *	0.21 ± 0.00 *	0.27 ± 0.00 *
Day 7 (g)	4.73 ± 0.05 *	1.35 ± 0.00 ^h^	0.15 ± 0.00 ^fh^	0.11 ± 0.00 *	0.21 ± 0.00 ^hi^	0.27 ± 0.00 *
Day 8 (h)	4.57 ± 0.05 *	1.335 ±0.00 ^gi^	0.153 ± 0.00 ^gi^	0.111 ± 0.00 ^i^	0.21 ± 0.00 ^gi^	0.27 ± 0.00 *
Day 9 (i)	4.30 ± 0.02 *	1.33 ± 0.00 ^hj^	0.15 ± 0.00 ^hj^	0.11 ± 0.00 ^h^	0.21 ± 0.00 ^ghj^	0.28 ± 0.00 *
Day 10 (j)	4.24 ± 0.02 *	1.32 ± 0.00 ^ik^	0.15 ± 0.00 ^ik^	0.11 ± 0.00 *	0.21 ± 0.00 ^ik^	0.28 ± 0.00 *
Day 11 (k)	4.16 ± 0.02 *	1.32 ± 0.00 ^jl^	0.16 ± 0.00 ^j^	0.12 ± 0.00 *	0.21 ± 0.00 ^jlm^	0.29 ± 0.00 *
Day 12 (l)	4.09 ± 0.02 *	1.31 ± 0.00 ^km^	0.16 ± 0.00 *	0.12 ± 0.00 ^m^	0.21 ± 0.00 ^kmn^	0.29 ± 0.00 ^m^
Day 13 (m)	4.05 ± 0.02 ^n^	1.30 ± 0.00 ^l^	0.16 ± 0.00 *	0.12 ± 0.00 ^ln^	0.21 ± 0.00 ^kln^	0.29 ± 0.00 ^ln^
Day 14 (n)	4.01 ± 0.01 ^mo^	1.28 ± 0.00 ^o^	0.16 ± 0.00 *	0.12 ± 0.00 ^m^	0.21 ± 0.00 ^lmo^	0.29 ± 0.00 ^mo^
Day 15 (o)	3.97 ± 0.01 ^n^	1.26 ± 0.00 ^np^	0.16 ± 0.00 *	0.12 ± 0.00 *	0.21 ± 0.00 ^np^	0.29 ± 0.00 ^np^
Day 16 (p)	3.92 ± 0.02 *	1.26 ± 0.00 ^oq^	0.17 ± 0.00 *	0.12 ± 0.00 *	0.21 ± 0.00 ^oq^	0.29 ± 0.00 ^o^
Day 17 (q)	3.82 ± 0.02 ^r^	1.25 ± 0.00 ^pr^	0.17 ± 0.00 *	0.13 ± 0.00 *	0.21 ± 0.00 ^prs^	0.30 ± 0.00 ^r^
Day 18 (r)	3.79 ± 0.02 ^qs^	1.24 ± 0.00 ^qs^	0.17 ± 0.00 *	0.13 ± 0.00 ^s^	0.21 ± 0.00 ^qs^	0.30 ± 0.00 ^q^
Day 19 (s)	3.76 ± 0.01 ^rt^	1.23 ± 0.00 ^rt^	0.17 ± 0.00 *	0.13 ± 0.00 ^rt^	0.21 ± 0.00 ^qrt^	0.30 ± 0.00 ^t^
Day 20 (t)	3.73 ± 0.01 ^s^	1.23 ± 0.00 ^su^	0.17 ± 0.00 *	0.13 ± 0.00 ^s^	0.21 ± 0.00 ^su^	0.30 ± 0.00 ^s^
Day 21 (u)	3.58 ± 0.04 *	1.22 ± 0.00 ^tv^	0.17 ± 0.00 *	0.13 ± 0.00 *	0.21 ± 0.00 ^t^	0.30 ± 0.00 *
Day 22 (v)	3.49 ± 0.03 *	1.21 ± 0.00 ^u^	0.17 ± 0.00 *	0.19 ± 0.00 *	0.21 ± 0.00 *	0.34 ± 0.00 *

V1—no yeast, fermented in glass vessels. V2—Fermol^®^ Candy, fermented in glass vessels. V3—Fermactive^®^ Sauvignon, fermented in glass vessels. V4—Fermol^®^ Candy, fermented in stainless steel tanks. V5—Fermactive^®^ Sauvignon, fermented in stainless steel tanks. Different superscript letters show that the fermentation moments do not have a statistically significant influence on organic acid concentration. *—indicates that the concentration of the identified organic acid is significantly influenced by the fermentation stage.

**Table 6:** Evolution of main organic acids during alcoholic fermentation of V5 variant (g/L).

Fermentation Stage	Tartaric Acid	Malic Acid	Lactic Acid	Acetic Acid	Citric Acid	Succinic Acid
Day 1 (a)	5.07 ± 0.02 ^ef^	1.87 ± 0.01 *	0.10 ± 0.00 *	0.10 ± 0.00 ^b^	0.20 ± 0.00 *	0.26 ± 0.00 *
Day 2 (b)	5.21 ± 0.03 *	1.82 ± 0.00 ^c^	0.10 ± 0.00 *	0.10 ± 0.00 ^a^	0.20 ± 0.00 *	0.29 ± 0.00 *
Day 3 (c)	5.16 ± 0.03 *	1.81 ± 0.01 ^b^	0.11 ± 0.00 *	0.11 ± 0.00 *	0.21 ± 0.00 *	0.30 ± 0.00 *
Day 4 (d)	5.11 ± 0.03 ^e^	1.79 ± 0.01 *	0.12 ± 0.00 *	0.12 ± 0.00 *	0.21 ± 0.00 *	0.30 ± 0.00 ^efg^
Day 5 (e)	5.07 ± 0.00 ^adf^	1.75 ± 0.02 *	0.13 ± 0.00 ^f^	0.15 ± 0.00 *	0.21 ± 0.00 *	0.30 ± 0.00 ^dfg^
Day 6 (f)	5.04 ± 0.02 ^ae^	1.70 ± 0.02 *	0.13 ± 0.00 ^eg^	0.15 ± 0.00 *	0.21 ± 0.00 *	0.30 ± 0.00 ^degh^
Day 7 (g)	5.00 ± 0.02 *	1.65 ± 0.02 *	0.13 ± 0.00 ^fh^	0.16 ± 0.00 *	0.21 ± 0.00 *	0.30 ± 0.00 ^defhij^
Day 8 (h)	4.48 ± 0.02 *	1.62 ± 0.01 *	0.13 ± 0.00 ^gi^	0.16 ± 0.00 *	0.21 ± 0.00 ^i^	0.30 ± 0.00 ^fgijk^
Day 9 (i)	4.440 ±0.02 ^j^	1.56 ± 0.01 *	0.13 ± 0.00 ^h^	0.17 ± 0.00 *	0.21 ± 0.00 ^hj^	0.30 ± 0.00 ^ghjk^
Day 10 (j)	4.41 ± 0.01 ^i^	1.53 ± 0.01 *	0.14 ± 0.00 *	0.17 ± 0.00 *	0.21 ± 0.00 ^ik^	0.31 ± 0.00 ^ghikl^
Day 11 (k)	4.36 ± 0.02 *	1.50 ± 0.01 *	0.14 ± 0.00 ^l^	0.18 ± 0.00 *	0.21 ± 0.00 ^j^	0.31 ± 0.00 ^hijlm^
Day 12 (l)	4.24 ± 0.03 *	1.47 ± 0.00 *	0.14 ± 0.00 ^km^	0.18 ± 0.00 *	0.21 ± 0.00 *	0.31 ± 0.00 ^jkmno^
Day 13 (m)	4.19 ± 0.00 ^n^	1.46 ± 0.00 ^n^	0.14 ± 0.00 ^ln^	0.19 ± 0.00 ^n^	0.21 ± 0.00 ^n^	0.31 ± 0.00 ^klno^
Day 14 (n)	4.15 ± 0.01 ^mo^	1.44 ± 0.00 ^mop^	0.14 ± 0.00 ^mo^	0.19 ± 0.00 ^mo^	0.21 ± 0.00 ^mo^	0.31 ± 0.00 ^lmo^
Day 15 (o)	4.14 ± 0.00 ^np^	1.43 ± 0.00 ^npq^	0.14 ± 0.00 ^np^	0.19 ± 0.00 ^n^	0.21 ± 0.00 ^np^	0.31 ± 0.00 ^lmn^
Day 16 (p)	4.11 ± 0.01 ^oq^	1.43 ± 0.00 ^noqr^	0.14 ± 0.00 ^o^	0.20 ± 0.00 *	0.21 ± 0.00 ^o^	0.31 ± 0.00 *
Day 17 (q)	4.10 ± 0.00 ^p^	1.43 ± 0.00 ^oprs^	0.15 ± 0.00 *	0.20 ± 0.00 ^v^	0.21 ± 0.00 *	0.32 ± 0.00 *
Day 18 (r)	4.06 ± 0.00 *	1.43 ± 0.00 ^pqst^	0.15 ± 0.00 *	0.20 ± 0.00 ^v^	0.21 ± 0.00 ^s^	0.33 ± 0.00 *
Day 19 (s)	3.81 ± 0.00 *	1.41 ± 0.00 ^qrt^	0.15 ± 0.00 *	0.21 ± 0.00 *	0.21 ± 0.00 ^rt^	0.33 ± 0.00 *
Day 20 (t)	3.76 ± 0.01 *	1.40 ± 0.00 ^rsu^	0.15 ± 0.00 *	0.21 ± 0.00 *	0.22 ± 0.00 ^s^	0.33 ± 0.00 *
Day 21 (u)	3.72 ± 0.01 *	1.39 ± 0.01 ^t^	0.16 ± 0.00 *	0.212 ± 0.00 *	0.22 ± 0.00 *	0.34 ± 0.00 ^v^
Day 22 (v)	3.64 ± 0.03 *	1.37 ± 0.01 *	0.17 ± 0.00 *	0.20 ± 0.00 ^qr^	0.22 ± 0.00 *	0.34 ± 0.00 ^u^

V1—no yeast, fermented in glass vessels. V2—Fermol^®^ Candy, fermented in glass vessels. V3—Fermactive^®^ Sauvignon, fermented in glass vessels. V4—Fermol^®^ Candy, fermented in stainless steel tanks. V5—Fermactive^®^ Sauvignon, fermented in stainless steel tanks. Different superscript letters show that the fermentation moments do not have a statistically significant influence on organic acid concentration. *—indicates that the concentration of the identified organic acid is significantly influenced by the fermentation stage.
